# Supplementary material for: Resolving the full spectrum of human genome variation using Linked-Reads
Source: Genome Res. 2019 Apr;29(4):635–45. doi: 10.1101/gr.234443.118 (PMC6442396; doi:10.1101/gr.234443.118)
Supplement: Supplemental Material [file supp_gr.234443.118_Supplemental_longranger-2.2.zip › longranger-2.2.tar.bz2!/lr2.2-public/lib/stan/cmdstan/stan/src/docs/talks/nyc-ml-meetup-2012/img/nuts-ess-1.pdf]

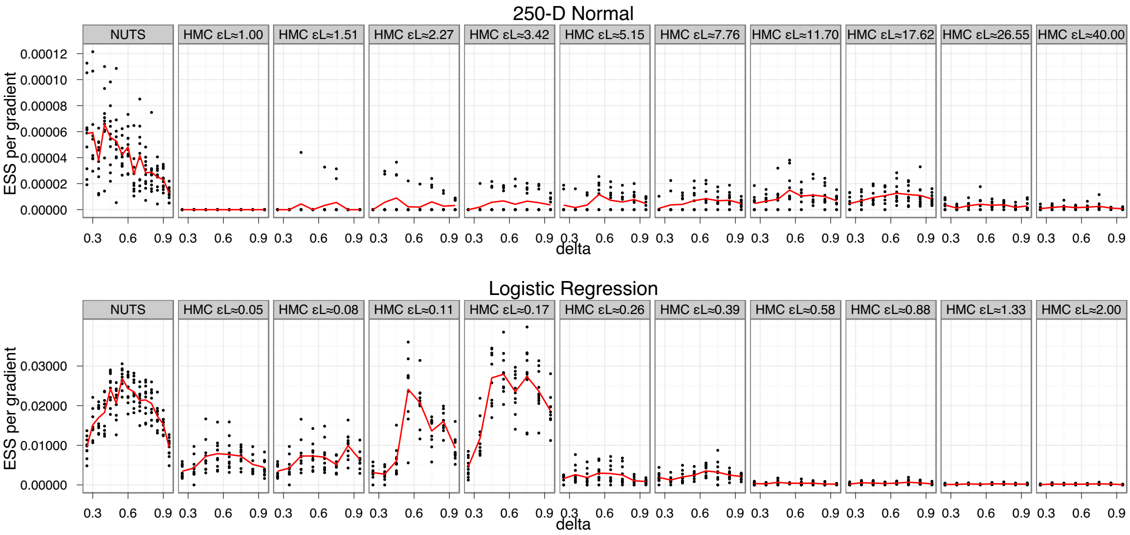
250-D Normal
NUTS
HMC εL≈1.00
HMC εL≈1.51
HMC εL≈2.27
HMC εL≈3.42
HMC εL≈5.15
HMC εL≈7.76
HMC εL≈11.70
HMC εL≈17.62
HMC εL≈26.55
HMC εL≈40.00
ESS per gradient
0.00012
0.00010
0.00008
0.00006
0.00004
0.00002
0.00000
0.3 0.6 0.9
delta
Logistic Regression
NUTS
HMC εL≈0.05
HMC εL≈0.08
HMC εL≈0.11
HMC εL≈0.17
HMC εL≈0.26
HMC εL≈0.39
HMC εL≈0.58
HMC εL≈0.88
HMC εL≈1.33
HMC εL≈2.00
ESS per gradient
0.03000
0.02000
0.01000
0.00000
0.3 0.6 0.9
delta
